# Multidrug-Resistant Tuberculosis of the Spine With Bilateral Psoas and Pre- and Paravertebral Abscesses in an Immunocompetent Indian Female With Multiple Adverse Drug Reactions: The World’s First Report

**DOI:** 10.7759/cureus.51835

**Published:** 2024-01-08

**Authors:** Sankalp Yadav

**Affiliations:** 1 Medicine, Shri Madan Lal Khurana Chest Clinic, New Delhi, IND

**Keywords:** pott's spine, multi-drug resistant tb (mdr tb), paravertebral abscess, prevertebral abscess, psoas abcess, cbnaat/ xpert/ rif assay, line probe assays (lpa)

## Abstract

Tuberculosis is prevalent in high-burden countries. Extrapulmonary drug-resistant tuberculosis is exceedingly rare. Simultaneous involvement of the spine with psoas muscles in the absence of pulmonary seeding with a drug-resistant strain of *Mycobacterium tuberculosis* in an adult female is never reported. A 33-year-old Indian female presented with complaints of chronic back pain for eight months. She was on antituberculous treatment for Pott’s spine for six months. The diagnosis was challenging due to the paucibacillary nature of the disease, and it required a high index of suspicion backed by radiometric investigations, liquid culture, a line probe assay, and cartridge-based nucleic acid amplification of the pus. Further, her treatment was associated with multiple adverse drug reactions like a rise of QTcF (QT corrected for heart rate by Fridericia's cube root formula) on the electrocardiogram, peripheral neuropathy, and abnormal behavior (decreased awareness and anger outbursts with restlessness), which were addressed by a team of experts including a cardiologist, a psychiatrist, a neurologist, and an infectious disease expert. She was managed conservatively, with an 18-month-long antituberculous treatment, which was stopped after consultation with an orthopedist at a nodal drug-resistant tuberculosis center with the advice to consult the orthopedic outpatient department; however, she was lost to follow-up.

## Introduction

In countries where tuberculosis is still prevalent, there is an imminent threat to the health of the people. Mycobacteria strains that are resistant to drugs have exacerbated the situation, necessitating prompt action for effective therapy [1]. As per the WHO's recently released Global Tuberculosis Report 2023, India contributes 27% of the total cases of tuberculosis, with 22% mortality worldwide [2].

In about 1-2% of all cases of tuberculosis, there is involvement of the skeletal system, which further constitutes about 10% of all extrapulmonary tuberculosis cases [3]. Though skeletal tuberculosis can affect any portion of the skeleton, the spine continues to be the most commonly affected (50%), followed by long bones [4].

*Mycobacterium tuberculosis* complex strains that are resistant to at least isoniazid and rifampicin are the cause of multidrug-resistant tuberculosis [5]. With 1.3 million cases of drug-resistant tuberculosis in 2018, India has the second-highest global burden of multidrug-resistant tuberculosis. A total of 2.8% of new cases and 12% of those that have already received treatment have multidrug-resistant tuberculosis. The incidence of multidrug-resistant tuberculosis and rifampicin-resistant tuberculosis in India was 135,000 [5,6]. Further, this incidence of spinal tuberculosis is about 10% of total tuberculosis cases [7].

Herein, a case of a 33-year-old Indian female is presented who came with complaints of chronic back pain even after undergoing antituberculous treatment for six months for drug-sensitive spinal tuberculosis, or Pott’s spine. Because of the paucibacillary character of the illness, the diagnosis was complex to establish and required an advanced level of suspicion verified through extensive laboratory and radiometric testing. She received conservative management, and the post-treatment evaluation showed a marked improvement.

## Case presentation

A 33-year-old married, non-diabetic Indian female belonging to a low socioeconomic background reported to the outpatient department with a backache for 18 months. This pain was insidious in onset, was intermittent for the first three months (relieved by taking over-the-counter analgesic diclofenac sodium), and subsequently became constant, affecting her daily routine. She was on antituberculous treatment (with a fixed-dose combination of isoniazid, pyrazinamide, rifampicin, and ethambutol for 56 days, followed by isoniazid, rifampicin, and ethambutol for 14 months) for sixteen months for drug-sensitive spinal tuberculosis, or Pott’s spine. As per the national guidelines, she was given an extension of antituberculous treatment after consultation with an orthopedist. She was advised bed rest at the time of the initiation of her treatment, but she was doing her household work. Also, she was irregularly visiting orthopedic and infectious disease outpatient departments for follow-ups.

There was no history of coughing, loss of weight, loss of appetite, night sweats, or fever. There was no history of trauma, heavy weight lifting, or any contact with tuberculosis. She also had no other history of any major medical or surgical intervention in the past. She was a homemaker with no history of substance misuse, incarceration, or overnight stays in shelters or camps for refugees.

A detailed clinical examination revealed an afebrile woman with a weight of 50 kg and a BMI of 21.6 kg/m^2^. Local examination revealed marked spinal tenderness of the dorso-lumbar spine with a significant limitation of spinal motion, i.e., during bending or getting up from her bed. She did not have respiratory symptoms, and her neurologic and cardiovascular examinations were normal.

The laboratory evaluation showed a hemoglobin of 10.5 g/dl and a total leucocyte count of 5800/mm3 with 71% polymorphonuclear cells and 19% lymphocytes. Erythrocyte sedimentation rate (first hour) and C-reactive proteins were, respectively, 63 mm and 21 mg/l. Induced sputum for acid-fast bacilli direct smear as well as mycobacterium culture were negative. Her HIV (I and II) test was also non-reactive.

**Figure 1:**
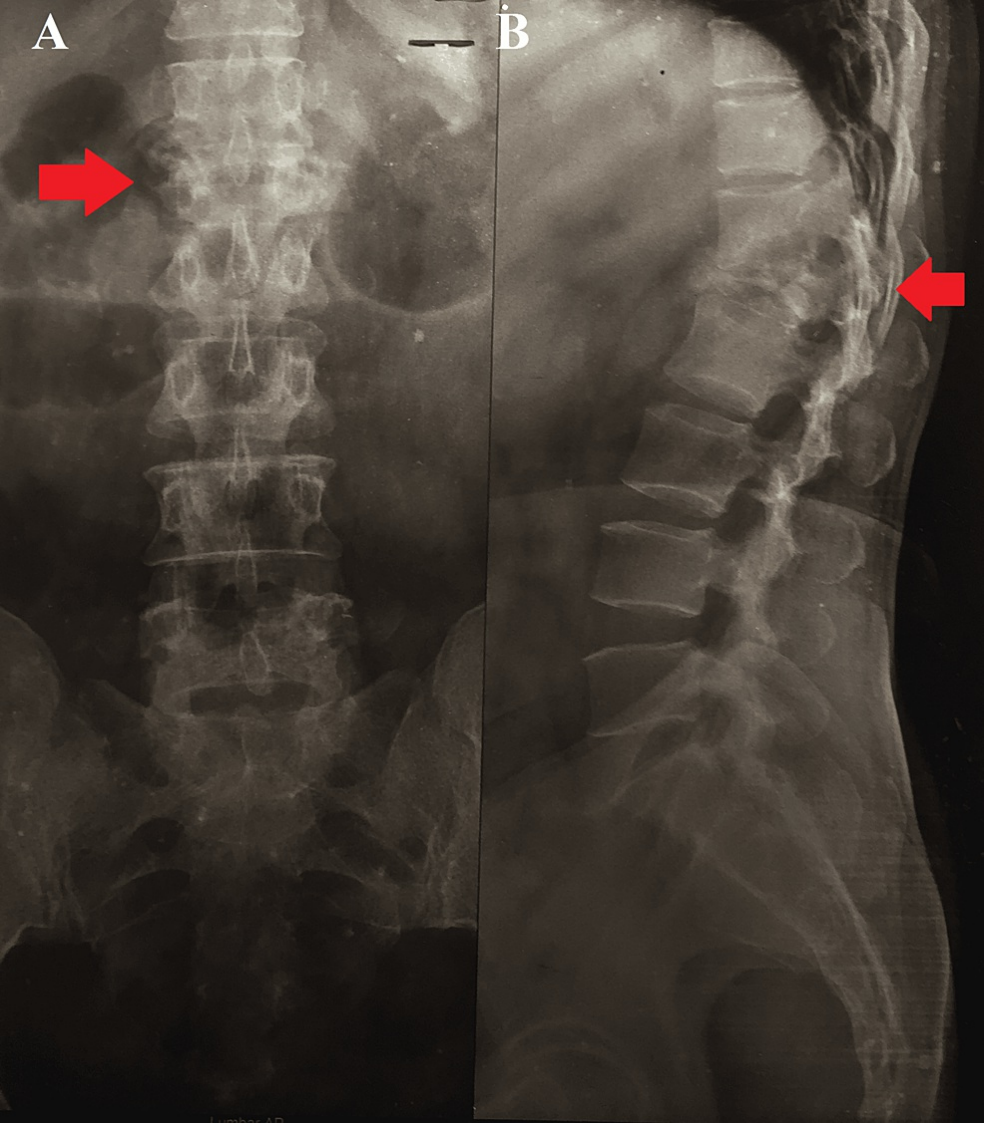
Plain radiograph of the lumbar region A: AP view, B: Lateral view

Further investigations, including a radiograph of the lumbar region, showed destruction at the lower shelf of L1 and the upper shelf of L2 (Figure 1).

**Figure 2:**
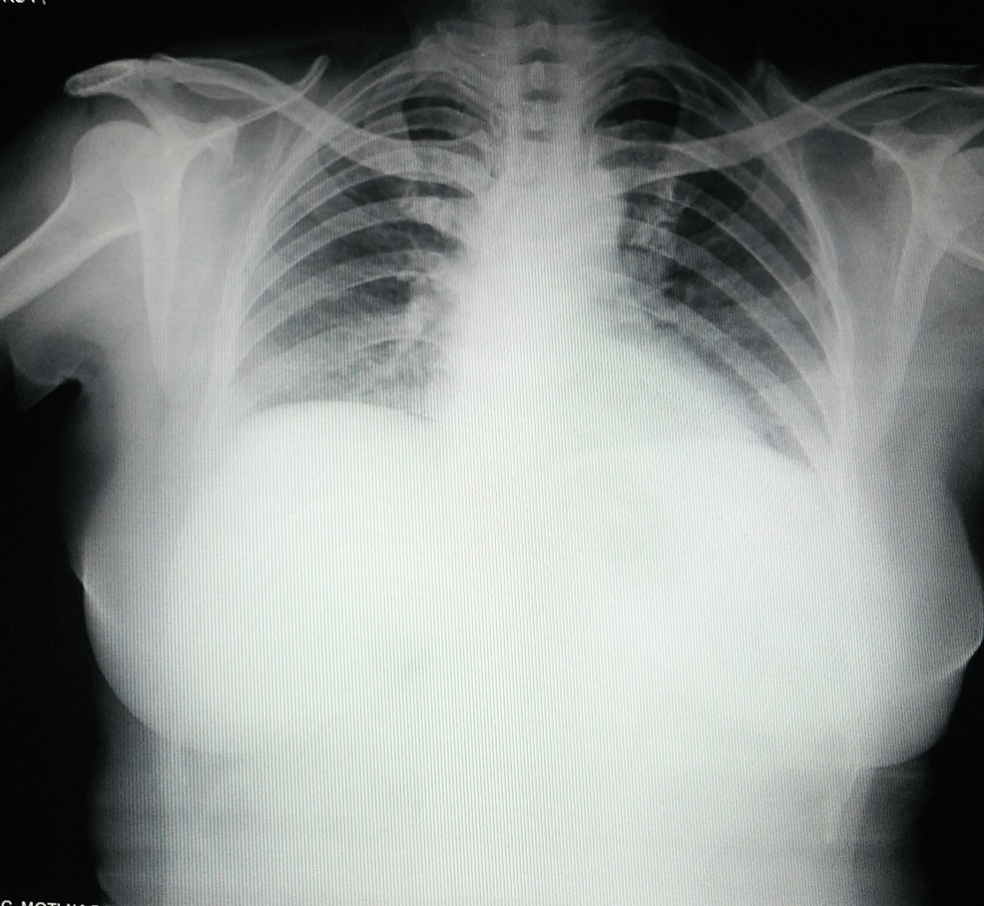
A normal chest radiograph

A chest radiograph was unremarkable (Figure 2).

**Figure 3:**
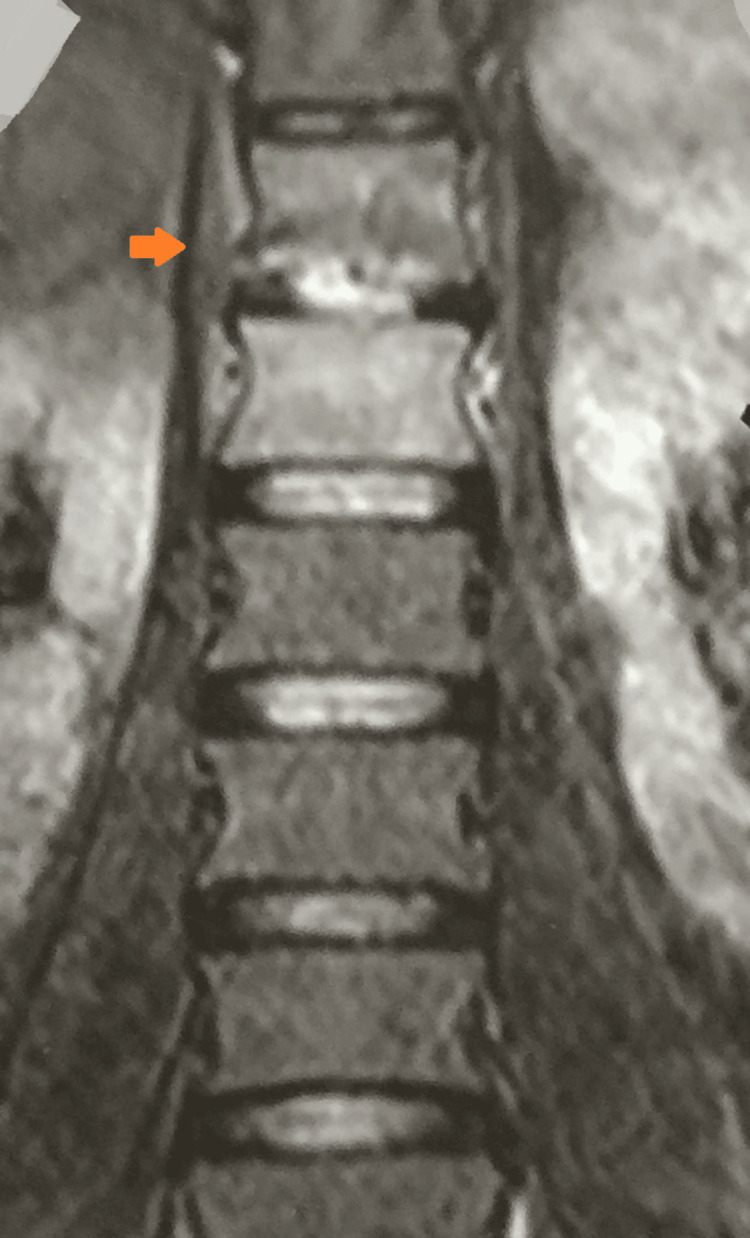
MRI of the dorso-lumbar spine showing bone marrow edema at D12 and L1 vertebral bodies with cortical destruction of the posterior part of D12 vertebral

An MRI of the dorso-lumbar spine done at the initiation of her antituberculous treatment sixteen months ago for drug-sensitive spinal tuberculosis showed bone marrow edema in the D12 and L1 vertebral bodies with cortical destruction of the posterior part of the D12 vertebral body. There were no prevertebral, paravertebral, or psoas collections (Figure 3).

**Figure 4:**
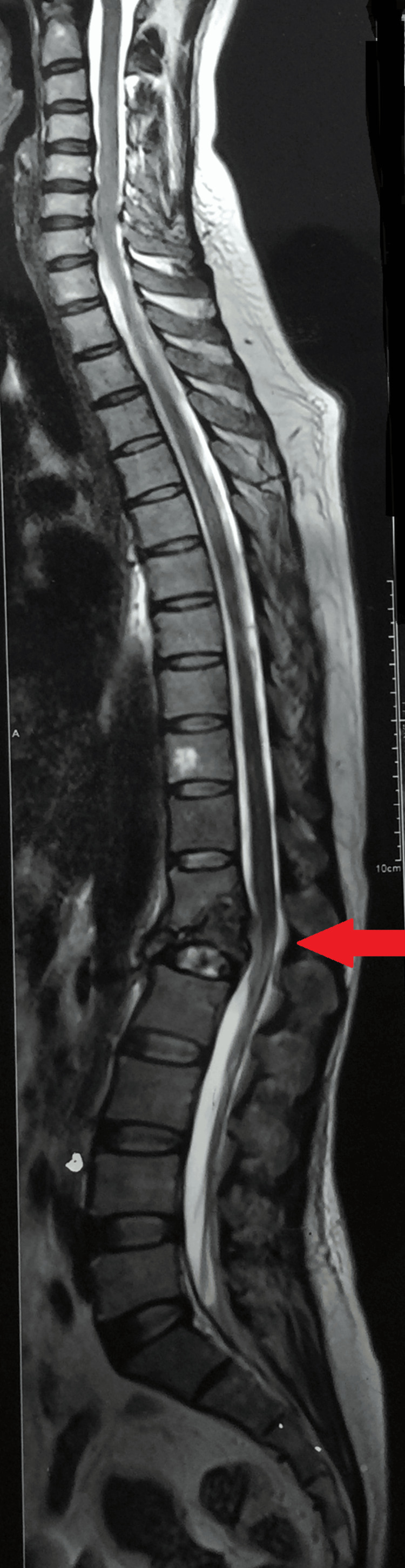
MRI of the dorso lumbar spine showing the collapse of the first lumbar vertebra with cortical destruction and marrow edema with partial collapse of D12 with loss of D12-L1 height with inferior endplate erosion

**Figure 5:**
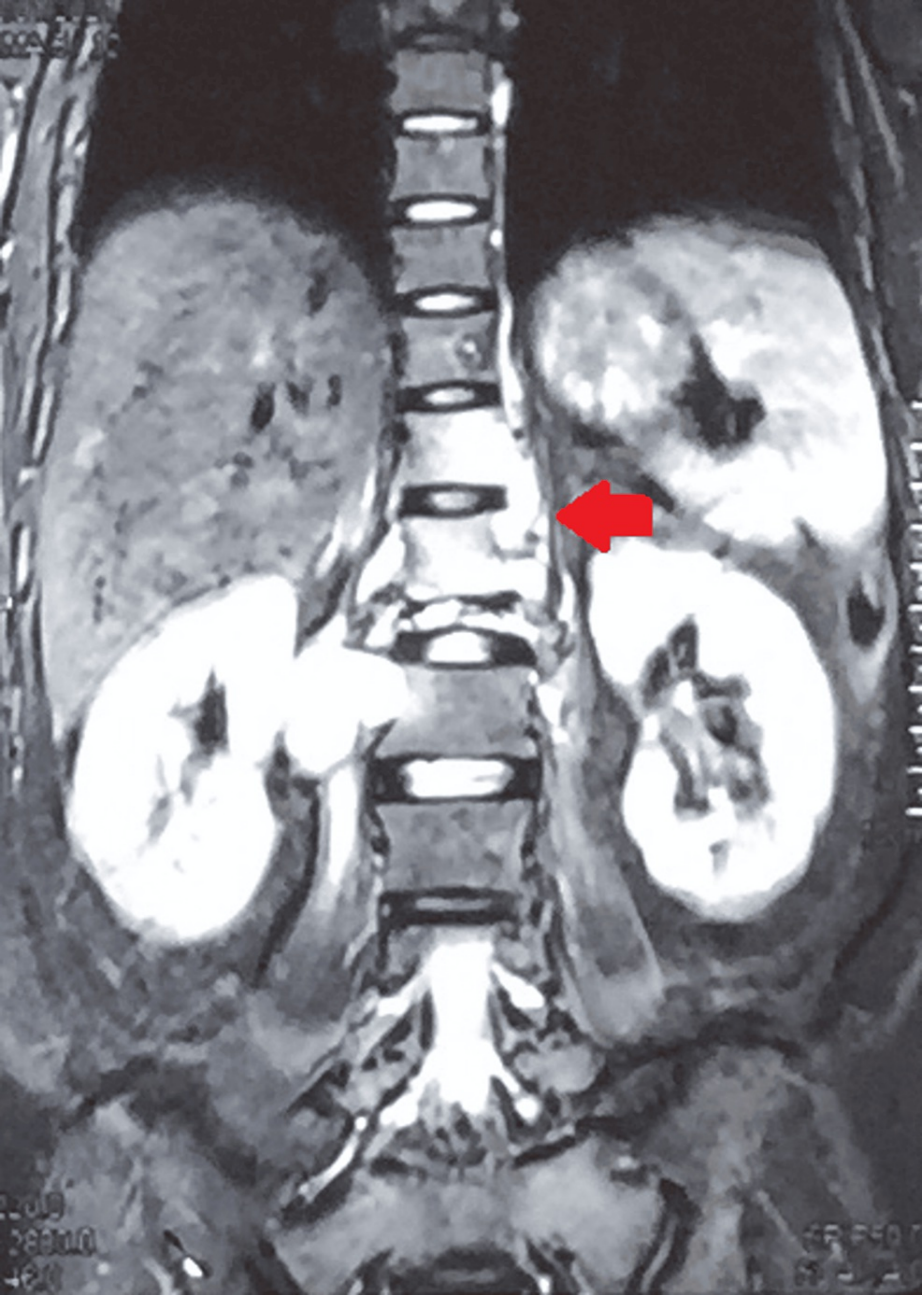
MRI of the dorso-lumbar spine with multiple tiny pre- and paravertebral collections

**Figure 6:**
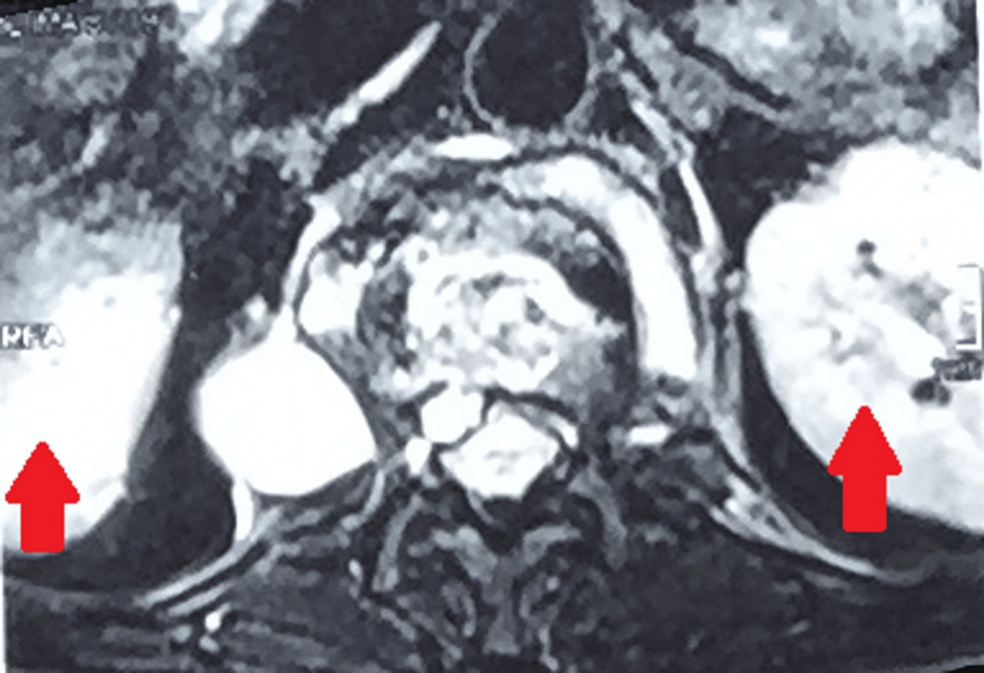
MRI of the dorso-lumbar spine showing bilateral psoas abscesses

A follow-up MRI of the dorso-lumbar spine at the time of presentation in the outpatient department after 16 months revealed a significant collapse of the first lumbar vertebra with cortical destruction and marrow edema. There was a partial collapse of D12 with a loss of D12-L1 height with inferior endplate erosion. Mild bone marrow edema was also seen in the D11 and L2 vertebral bodies. Multiple tiny pre- and paravertebral collections were noted at these levels. There were large psoas abscesses on the right (27 x 57 mm) and left (25 x 12 mm). Mild kyphosis was also seen at these levels. Mild retropulsion of the L1 vertebral body was seen with a small anterior epidural collection (Figures 4, 5, 6).

Due to the deterioration of her condition evident clinically and on the MRI, a detailed workup with suspicion of drug resistance was done. An ultrasound-guided surgical debridement with removal of about 50 cc of pus was done on the psoas muscles. The direct smear of pus for acid-fast bacilli was negative. However, her cartridge-based nucleic acid amplification of the pus was positive with medium detection of *Mycobacterium tuberculosis *with resistance to rifampicin. Another sample sent for liquid automated culture was suggestive of the growth of *Mycobacterium tuberculosis* with resistance to rifampicin and isoniazid. A line probe assay after inoculation into liquid culture was suggestive of *Mycobacterium* *tuberculosis* with resistance to rifampicin and isoniazid (high level to the katG gene). The results of second-line drug susceptibility testing were not suggestive of any other additional drug resistance. Hence, she was diagnosed with multidrug-resistant tuberculosis of the spine with bilateral psoas and pre- and paravertebral abscess; a pretreatment evaluation was done to initiate an all-oral longer regimen as per the national guidelines (Table 1).

**Table 1:** All-oral longer regimen per the national guidelines OD: Once a day; AD: Alternate day

Drug	Route of administration	Dose	Duration
Bedaquiline	Per oral	400 mg X OD and then 200 mg AD	24 weeks (2+22)
Linezolid	Per oral	600 mg X OD, followed by 300 mg X OD	18 months (6+12)
Levofloxacin	Per oral	1000 mg X OD	540 days
Clofazimine	Per oral	100 mg X OD	540 days
Cycloserine	Per oral	750 mg X OD	540 days
Pyridoxine	Per oral	100 mg X OD	540 days

She tolerated the treatment well initially (one week), but later had multiple adverse drug reactions, including a rise of QTcF on the electrocardiogram (after two weeks, at regular follow-up), peripheral neuropathy (at sixth-month follow-up), and abnormal behavior (decreased awareness and anger outbursts with restlessness) at 9-month follow-up. As a result, she was advised to have regular electrocardiograms and serum electrolyte levels were assessed for her bedaquiline. For peripheral neuropathy, linezolid was replaced with tablet delamanid (100 mg twice daily) in the sixth month of treatment after a nerve conduction study. A psychiatrist's opinion and a neurologist’s opinion were taken, which assessed the role of cycloserine; however, her symptoms subsided post-administration of tablet clonazepam (0.5 g) for five days. After these modifications to her treatment, there were no remarkable adverse drug reactions, and she completed her treatment.

**Figure 7:**
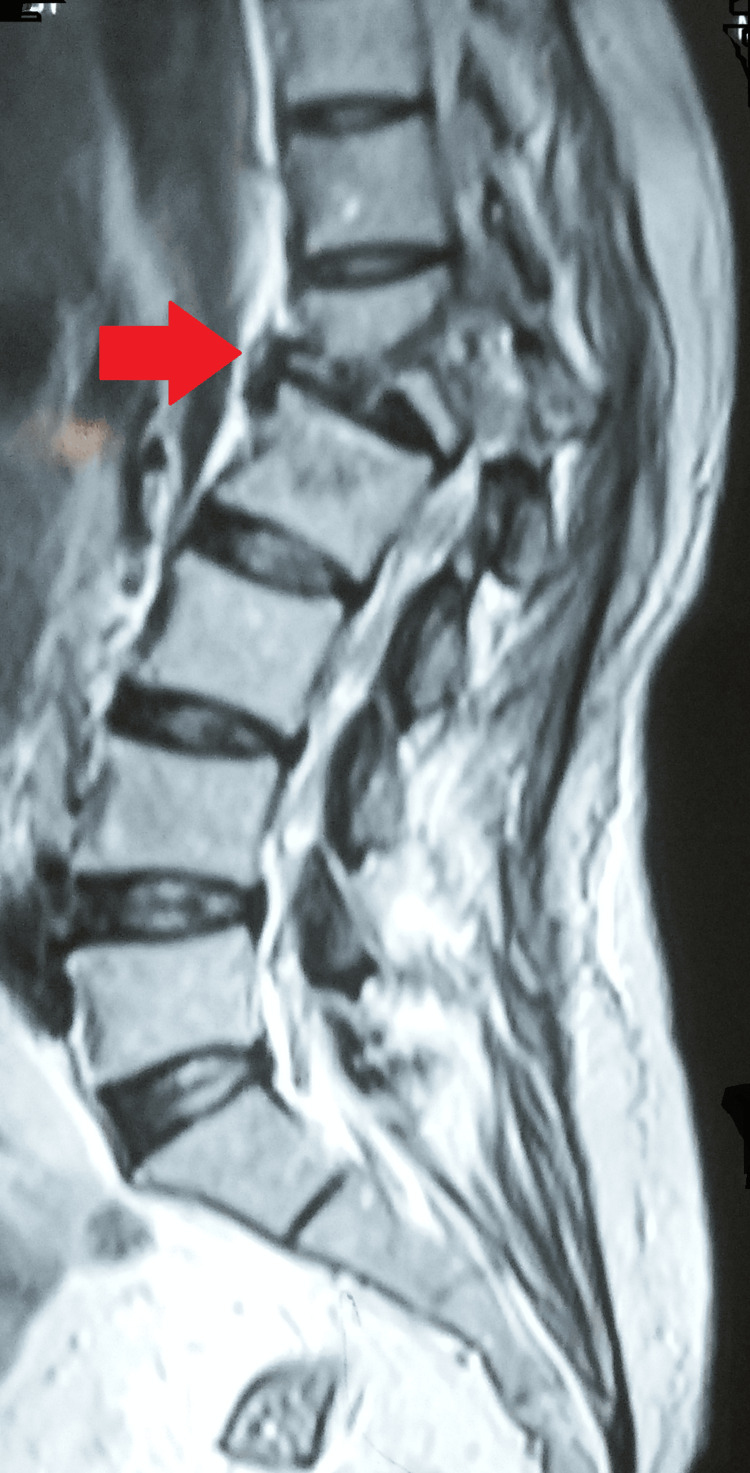
MRI of the dorso-lumbar spine showing a partial collapse of the D12 vertebral body and a significant collapse of the L1 vertebral body with a loss of the D12-L1 height

A post-treatment follow-up MRI of the dorso-lumbar spine in the eighteenth month showed a partial collapse of the D12 vertebral body and a significant collapse of the L1 vertebral body with a loss of the D12-L1 height. Minimal bone marrow edema was seen in the L1 vertebral body. There were no prevertebral, paravertebral, or psoas collections showing a near-complete resolution of active disease. Early spondylo-disco-degenerative changes with no obvious disc bulge or protrusion were evident (Figure 7).

She was advised to continue consultation in the orthopedic outpatient department with regular follow-ups in the infectious disease unit. However, she was lost to follow-up.

## Discussion

Multidrug-resistant tuberculosis is a significant threat to public health globally. It’s a rare occurrence, especially in immunocompetent individuals [8]. A case of multidrug-resistant tuberculosis of the spine is rare, and this presentation with bilateral psoas and pre- and paravertebral abscesses with multiple adverse drug reactions is never reported in an adult female.

Multidrug-resistant tuberculosis is a man-made problem mainly attributed to poor drug therapy, a lack of knowledge on the part of the prescribing physician or surgeon, difficulty for low-income patients to obtain drugs due to lack of funds or social insurance, frequent shortages of second-line antituberculous drugs due to poor management and/or financial constraints, the use of drugs or fixed drug combinations containing drugs with unknown bioavailability, lack of motivation at the start of treatment, and inadequate self-administration of drugs without direct supervision during the intensive phase of therapy [7].

The clinical manifestation of tuberculosis in the spine varies greatly. The beginning of spinal tuberculosis is typically subtle, and the illness advances slowly. From the time of symptom onset, the diagnosis interval can range from a few weeks to several years. There are two types of spinal tuberculosis: complicated and uncomplicated. Patients with complicated tuberculosis often have neurological deficiencies, deformities, and instability. A diagnosis of uncomplicated spinal tuberculosis is one that occurs before these problems manifest. Of all the symptoms, back pain is the most prevalent, as seen in the present case. In the active stage, bone inflammation is the main cause and might sporadically manifest as radicular symptoms. Constitutional symptoms of tuberculosis, such as loss of appetite, fever, night sweats, loss of weight, and malaise, are less frequently associated with spinal tuberculosis [9].

Diagnosis of multidrug-resistant tuberculosis of the spine is an arduous task, mainly due to challenges in obtaining the samples for drug susceptibility testing. Furthermore, since spinal multidrug-resistant tuberculosis is a paucibacillary illness, there is very little chance of both bacterial growth and culture sensitivity, even in the event that an appropriate sample is obtained [10]. Besides, the diagnosis of spinal tuberculosis is frequently delayed because of the existence of neurological consequences and spinal deformities [11]. In areas where spinal tuberculosis is endemic, the diagnosis is typically made after a mean delay of six to eight months. Drug-resistant cases of spinal tuberculosis are suspected when the patient exhibits no response to antituberculous drugs for at least six months, which further delays the diagnosis of drug-resistant cases of tuberculosis in the spine [11]. However, with the availability of universal drug susceptibility testing, these delays can be avoided at the health facility level [12].

MRI findings, along with clinical evidence, are used to diagnose spinal tuberculosis. Deep-seated paucibacillary lesions are the hallmark of spinal tuberculosis. However, to establish a definite diagnosis, tissue or pus samples need to be collected by invasive procedures and the organism needs to be identified using Ziehl-Neelsen stain, histopathology, cartridge-based nucleic acid amplification, line-probe assay, and culture, as in the present case [10].

Multidrug-resistant tuberculosis of the spine treatment is a complex, hard, expensive, and time-consuming procedure that requires the experience and skills of a team involving an infectious disease expert, an orthopedist, and a counselor [7]. Management is mainly conservative with second-line antituberculous drugs. In India, the WHO-recommended all-oral longer treatment is indicated as per the national guidelines [12]. However, these second-line antituberculous drugs are associated with a number of adverse drug reactions as reported in this case. Several adverse drug reactions are asymptomatic for example the QTcF prolongation seen in this case was asymptomatic and found only on routine follow-up. Tingling and numbness are common in patients on second-line antituberculous drugs and are progressive in some cases, but it is only after a nerve conduction study that the management was changed with the omission of linezolid and the addition of delamanid. Cycloserine-induced insomnia, mania, psychosis, and suicidal ideation are available in the literature [13,14]. Surgical debridement is indicated for obtaining samples of the pus and tissues from the deep-seated paucibacillary lesions; however, it has the added advantage of reducing the bacterial load from the lesion [11]. Nevertheless, surgical intervention is indicated in cases of progressive neurological deficit, progressive spinal deformity, or failure of conservative treatment [3].

Reports of multidrug-resistant tuberculosis of the spine are available in the literature as research studies [7,10]. Pawar et al. were the first to report the clinical characteristics of 25 multidrug-resistant tuberculosis of the spine patients along with their drug susceptibility patterns. In their study, about 50% of the patients had adverse drug effects [7]. Similarly, Saodekar reported 80 patients in his hospital-based prevalence with a study duration of three years. He mentioned that early detection of multi-drug resistant spinal tuberculosis is essential to avoid consequences arising due to inappropriate antituberculous chemotherapy and to achieve the desired results [10]. In a study from China from 1999-2015, 272 patients with multidrug-resistant tuberculosis of the spine were assessed. The authors also emphasized early detection and management of these rare cases [15].

As detailed here, the diagnosis of multidrug-resistant tuberculosis of the spine is a tough ask, but prompt management is imperative as serious social and economic issues can arise from spinal tuberculosis, which frequently results in irreparable neurological damage, including paralysis [15].

## Conclusions

A case of a young female with no pulmonary involvement is presented. The diagnosis was difficult due to the paucibacillary nature of the disease, and it required a high index of suspicion backed by radiometric investigations, liquid culture, and cartridge-based nucleic acid amplification of the pus to establish the same, especially in a high-burden country. She was initiated on second-line antituberculous chemotherapy with the resolution of abscesses and no progression of disease after an assessment by the treating orthopedist. Prompt diagnosis and treatment maximized the prognosis for tuberculosis of the spine, as seen in this case. Besides, a high level of clinical skepticism should be applied to people who seem to have persistent back pain, even in the absence of neurological signs and indicators.
